# Synthesis and Optimization of a Free-Radical/Cationic Hybrid Photosensitive UV Curable Resin Using Polyurethane Acrylate and Graphene Oxide

**DOI:** 10.3390/polym14101959

**Published:** 2022-05-12

**Authors:** Lijie Huang, Yanan Wang, Zhehao Wei, Xiaoxue Han, Qi Mo, Xiyue Wang, Yishan Li

**Affiliations:** 1 College of Light Industry and Food Engineering, Guangxi University, Nanning 530004, China; wangyn0214@163.com (Y.W.); hxx1134414215aa@163.com (X.H.); moqigx@163.com (Q.M.); usamiwang@163.com (X.W.); ysli0904@163.com (Y.L.); 2 Guangxi Bossco Environmental Protection Technology Co., Ltd., Nanning 530007, China; wzh345495625@163.com

**Keywords:** photosensitive resin, orthogonal experiments, polyurethane acrylates, graphene oxide, tensile strength, gelation rate, volume shrinkage, UV curable resin, free radical polymerization, epoxy resin

## Abstract

Cost-effective, practical, and efficiently performing photosensitive resin composite materials are essential, as the current materials are expensive, lack better alternatives, and do not meet 3D printing standards. In this study, based on orthogonal experiments for photosensitive resin curing, we prepared a free-radical/cationic hybrid photosensitive UV cured resin (UVR) using acrylic ester and epoxy resin as the prepolymers, tripropylenediol diacrylate (TPGDA) as the active diluent, and triaryl sulfonium salt (I-160) and 2,2-dimethyl-α-hydroxy acetophenone (1173) as the photoinitiators, in the optimized formula of acrylic-ester:epoxy-resin:TPGDA:I-160:1173 = 37.5:37.5:20:2.5:2.5. Further, we investigated the effects of polyurethane acrylates (PUA) and Graphene oxide (GO) on the surface morphology, chemical structure, hydrophobicity, mechanical strength, and gelation rate of the hybrid resin. We observed that 20% PUA improved tensile strength to the maximum of 36.89 MPa from 16.42 MPa of the unmodified hybrid resin, whereas 1% GO reduced volume shrinkage to the minimum of 2.89% from 3.73% of the unmodified hybrid resin. These photosensitive resins with higher tensile strength and lower volume shrinkage can be used to synthesize high performance functional materials in the future.

## 1. Introduction

Three-dimensional (3D) printing commonly employs light curing as a molding method. Light curing technology has rapidly advanced in recent years [1]. In this process, a photosensitive resin undergoes stacking and curing upon irradiation with ultraviolet light, depending on the modeling shape [2]. Owing to its high efficiency and precision, flexibility, and sustainability, this novel light curing 3D printing technology is widely used in diverse materials such as coatings, printing inks, dental composites, and intelligent materials [3,4,5,6,7]. Light curing printing technology depends on the use of a high-performance photosensitive resin material. Photosensitive resins are widely used in flexible electronic devices, biological tissue engineering applicationics, and metamaterials [8,9,10] and can be classified into free radical and cationic photosensitive resins depending on their polymerization mechanism [11]. The active center of the free radical photoinitiator [12] decomposes under UV irradiation, which activates the double bond in the prepolymer molecule and forms active monomer free radicals. The active polymer chain continues to grow by the addition of these free radical monomers to finally form a solid macromolecular product [13]. The advantages of free radical photosensitive resins include their high photosensitivity, high curing speed, and low cost; however, these resins are highly susceptible to the inhibition of polymerization by oxygen during curing, such that any part of the resin in contact with oxygen is poorly cured and exhibits high volume shrinkage [14,15]. On the contrary, the cationic curing system is unaffected by oxygen, does not need inert gas protection, and has a high degree of curing; however, it is expensive and has a low curing rate. In addition, it is difficult to regulate the properties of the cured products, and the curing conditions need to be strictly controlled at low temperatures without water [16]. Therefore, a hybrid photosensitive resin that combines the characteristics of the free radical and cationic systems and produces a synergistic effect has been proposed [17,18]. This system can effectively overcome the difficulty of curing a single system by integrating the advantages of the two systems, compensate for the individual defects in performance, and achieve the effect of 1 + 1 > 2. In addition, interpenetrating network structures (IPNs) [19,20] may be formed during hybrid light curing, leading to inclusion and synergy, which improves the curing speed, weakens the influence of oxygen polymerization inhibition, and significantly reduces the shrinkage, Therefore, scholars are increasingly beginning to study hybrid photosensitive resins to combine the rapid curing rate of free radical photopolymerization and the good mechanical properties obtained by cationic polymerization [21,22,23].

IPNs consist of two or more chemically crosslinked permeable polymer networks and can be formed by polymerizing a mixture of multi-functional monomers in different ways [19,20,21,22,23,24]. Free radical polymerization is typically used to form acrylic resins [25], including epoxy acrylic resin [26], polyurethane acrylic resin [27], and polyester acrylic resin. Conversely, cationic polymerization typically forms epoxy resins [28,29] and vinyl ether resin with an epoxy or a vinyl ether group. Biwu et al. [30] used a mixture of acrylate resins as raw materials in the synthesis of a photosensitive resin for an epoxy acrylate hybrid system., which exhibited a shrinkage and crimp of less than 2.00% and 8.00%., respectively. Guanghong [31] et al. prepared a free radical/cationic hybrid photosensitive resin using a mixture of epoxy resin (epon828) and polyurethane acrylate (rj429), which exhibited a tensile strength of 6.60 MPa and a volume shrinkage and warpage of 3.986% and 3.62%, respectively. Zijun [19] blended an epoxy resin, acrylic resin, free radical, and cationic photoinitiator to obtain an IPN epoxy composite with a complex 3D structure and excellent toughness and ductility. Ting [32] et al. synthesized a hybrid system containing an epoxy acrylic acid, epoxy resin, and a photoinitiator, to which modified calcium sulfate whisker (CSW) was added as an inorganic filler. With a CSW content of 7%, a maximum tensile strength 27.81 MPa was obtained. Škola et al. [33] added free radical and cationic machine-polymerized adhesives to a mixture of acrylate, epoxide, and oxyheterocyclobutane prepolymers while varying the mixing ratio of the adhesives and monomers to produce a free-radical/cationic hybrid photosensitive resin with superior mechanical properties and conversion.

In this study, a free-radical/cationic hybrid photosensitive UV cured resin was prepared by a combination of cationic and free radical polymerization for SLA 3D printing. Polyurethane acrylates (PUA) and Graphene oxide (GO) modifiers were used to obtain a practical photosensitive resin with excellent mechanical properties. The effects of this modification on the mechanical properties, volume shrinkage, and gelation rate of the hybrid photosensitive resin were investigated. This novel photosensitive resin can be used to synthesize packaging materials for cushioning and intelligent materials. 

## 2. Materials and Methods

### 2.1. Materials

The materials used in this study are summarized as follows: 3,4-epoxy cyclohexyl methyl-3,4-epoxy cyclohexyl, in the pure state (Huaxin New Material Co., Ltd. Huizhou, China); PUA and TPGDA, in the pure state (Jiazhong Chemical Technology Co., Ltd., Nanjing, China); triaryl sulfonium salt (I-160), in the pure state (Huaxin New Material Co., Ltd., Huizhou, China); 2-Dimethyl-α-Hydroxy Acetophenone (1173), industrial-grade form (Yinchang New Material Co., Ltd., Shanghai, China); acetone, in the pure state (Sinopharm Group Chemical Reagent Co., Ltd., Beijing, China); and Powdered GO (99 wt%, Figure S1), industrial-grade form (Zhongsen Pilot Technology Co., Ltd., Shenzhen, China).

### 2.2. Methods

#### 2.2.1. Preparation of the Free Radical/Cationic Hybrid Photosensitive UV Cured Resin (UVR)

Orthogonal experiments were designed in which the mass ratio of acrylate to epoxy resin (A), mass fraction of the active diluent in the total system (B), and mass fraction of the photoinitiator in the total system (C) were considered as contributing factors to the tensile strength, volume shrinkage, and gelation rate of the resin. The level of each factor level (Table 1) and the orthogonal table L9 (34) were selected without considering the interaction between the factors. The orthogonal experimental scheme is shown in Table 2.

As shown in Table 2, each component with a certain mass (1173:I-160 = 1:1) was weighed on an analytical balance, placed in a beaker wrapped in tin foil, magnetically stirred for 30 min at 300 rpm, and then ultrasonicated in an ice bath for a further 30 min. The resultant product was stored in the dark for later use.

#### 2.2.2. Preparation of PUA Modified Photosensitive UV Cured Resin (PUA-UVR)

The optimized free radical cationic hybrid photosensitive resin was modified by adding different mass fractions of PUA (10, 15, 20, and 25%) to the free radical cationic hybrid photosensitive resin. These were subjected to magnetic stirring and ultrasonic shock defoaming for 30 min each and stored in the dark for later use.

#### 2.2.3. Preparation of GO Modified Photosensitive UV Cured Resin (GO-UVR)

The optimized free-radical/cationic hybrid photosensitive resin was modified by the addition of various mass fractions of GO (0.2, 0.5, 0.7, and 1%). The blends were magnetically stirred at 45 °C for 30 min, followed by an ultrasound ice bath ultrasound for 1 h. Blends devoid of bubbles and evenly dispersed GO-UVR were obtained. These were obtained, sealed, and stored in the dark for later use.

### 2.3. Measurement and Characterization

#### 2.3.1. Fourier-Transform Infrared (FTIR) Spectroscopy

Fourier-transform infrared spectroscopy in the attenuated total reflectance mode (FTIR-ATR) was used to detect the changes in the functional groups of the PUA-modified, GO-modified, and unmodified hybrid photosensitive resins before and after the reaction using a Tensor Ⅱ spectrometer (Bruker Technology Co., Ltd., Madison, WI, USA). Each sample was scanned for 16 s over the wavenumber range of 400–4000 cm^−1^.

#### 2.3.2. Contact Angle Measurement

Water contact angles of the PUA-modified, GO-modified, and unmodified free-radical/cationic hybrid photosensitive resins were measured to determine their hydrophobicity. At 23 °C, the contact angle on the surface of the solidified film was measured by the hemispheric method using a Clutz contact angle measuring instrument (German Kruss equipment company, Hamburg, Germany). A fine needle was controlled to drop water on the surface of the horizontally placed sample, and the contact angle between the water droplet and the solidified film was recorded for 1 s. The contact angle data were fitted by a computer.

#### 2.3.3. Apparent Morphology

After the corresponding photosensitive resin was quenched with liquid nitrogen, the cross section of the material was observed by a scanning electron microscope (SEM, Phenom Inc., Amsterdam, The Netherlands).

#### 2.3.4. Thermal Stability 

The thermal stability of the resins was examined to measure the effect of temperature on the deformation of the cured product. Before the test, the sample was dried in a blast drying oven at 45 °C for 24 h. The solidified product was reduced to fine particles, following which a sample (10 mg) was transferred into an alumina crucible and analyzed using a synchronous thermal analyzer (Discovery TGA 55, Newcastle, DE, USA). The test was carried out under nitrogen protection across a temperature range of 30–600 °C at a heating rate of 10 °C/min.

#### 2.3.5. Crystallinity 

The crystallinity of the resins was measured by X-ray powder diffraction (XRS, SAXS nanostart X-ray, Bruker Technology Co., Ltd., Karlsruhe, Germany). The scanning speed was 8°/min and the 2θ range was 4°–80°.

#### 2.3.6. Mechanical Structure 

Maximum tensile stress, elongation at break, and material deformation resistance were determined by tensile tests conducted on a universal material testing machine (Instron Corporation, Boston, MA, USA).

A cured sample (100 mm × 10 mm × 1 mm) was dried in an air-drying oven at 45 °C for 24 h. The dimensions were selected based on similar values in various experimental studies on the mechanical properties of polymers [34,35,36]. The samples were polished with sandpaper to ensure no cracks or other defects on the surfaces. The average value of triplicate measurements is reported. The thickness of the sample within the standard distance was measured with a digital caliper. The standard distance and the rate were set to 50 mm and 10 mm/min, respectively, and an average of three parallel tests is reported.

#### 2.3.7. Volume Shrinkage Determination

The volume shrinkage test was performed to determine the volume change in the material post-curing. Distilled water was used as the reference, and the density of the resin before and after curing was measured to obtain the volume shrinkage using a previously described method employed by Zhengzhi et al. [37].

Liquid density values were calculated as follows:

The weight of a dry specific gravity flask, m g, was measured using an analytical balance. Then, the flask was filled with distilled water and placed in a water bath at 25 °C. After the temperature of the liquid in the flask stabilized at 25 °C, the flask was plugged, and any spillage from the capillary was wiped with a filter paper. Simultaneously, the outside of the flask was dried, and its weight was measured, m_0_ g, following which the flask was emptied, dried, and filled with the corresponding resin solution. The same procedure was repeated for the resin solution to obtain the weight of the flask, m_1_ g. The density (ρ_1_) of the resin solution was calculated as per the equation:

(1) $$\rho_{1}=\frac{m_{1}-m}{m_{0}-m}\rho_{0}$$

where ρ_0_ = 0.97705 g/cm³ is the density of water at 25 °C.

Solid density values were calculated as follows:

The mass of the cured product was weighed, m_2_ g, and the solidified material was placed into the specific gravity flask, which was filled with distilled water at 25 °C. The flask was corked, the outside of it was dried with a filter paper, and its weight m_3_ g was measured. The density (ρ_2_) of the solidified material was obtained using the equation:

(2) $$\rho_{2}=\frac{m_{2}}{m_{0}+m_{2}-m_{3}}\rho_{0}$$

Finally, volume shrinkage was calculated using the equation:

(3) $$S_{V}=\frac{\rho_{2}-\rho_{1}}{\rho_{2}}\times 100\%$$

#### 2.3.8. Gel Rate Determination

The gelation rate test was used to measure the degree of cure in the photosensitive resin. A certain amount of photosensitive resin was cast in a polytetrafluoroethylene mold and cured for 2 min under an ultraviolet light source. The weight of the cured sample, M_1_ g, was measured, and the sample was completely immersed in acetone, during which time the residual uncured resin was removed. After 24 h, the sample was taken out. Then, it was placed for 4 h in a drum wind drying oven at 45 °C, and the dried solid resin was weighed as M_2_ g. The gelation rate was calculated using the equation:

(4) $$\mathrm{Gelation}\;\mathrm{rate}\left(\%\right)=\frac{M_{2}}{M_{1}}\times 100$$

#### 2.3.9. Rheological Properties

The rheological properties of the resin were investigated using the modular rheometer workstation (HAAKE mars4, Waltham, MA, USA). The measurement mode was the shear rate mode, and the measurement temperature and viscosity test error were 25 °C and ±1%, respectively.

## 3. Results and Discussion

### 3.1. Orthogonal Reaction Analysis

The results of orthogonal experiments are shown in Table 3. Table 4 shows the Range analysis results, in which k_1_ is the average of all the results of one of the three factors at level 1, and R refers to the range k_1_–k_3_. Because the optimum combination obtained by the analysis of individual indexes is generally inconsistent, the combined influence of the factors on the indicators must be considered to obtain an accurate optimized combination. The significance of each factor was determined by the analysis of variance (ANOVA) method (Table 5).

In the F-tests, a level of significance (α) of 0.05 indicates a 95% confidence level, i.e., the critical value F is deemed significant. If F > FA, the effect of this factor on the corresponding indicators is significant.

We integrated both range and variance analyses and selected the optimized combination of using orthogonal experiments. Factor A has the most significant influence on tensile strength, whereas its effect on volume shrinkage ranks second. The optimal level of both of these factors is A1. Factor A has the least significant effect on the gelation rate; thus, it is a secondary factor. Therefore, the optimized level of factor A is A1. Factor B has the most significant influence on volume shrinkage, and the optimal level is B1. The effect of factor B on both tensile strength and gelation rate is ranked second, and, thus, the optimal level is B2. Factor B has a highly significant influence on volume shrinkage. The values of tensile strength and gelation rate of the optimal level B1 are 2.23% and 0.65%, respectively, less than those of B2; however, the volume shrinkage of B1 is significantly higher (by 17.59%) than that of B2. Therefore, the optimized level of factor B is B1. Factor C only had a significant effect on gelation rate; therefore, we selected C3. Finally, the optimal horizontal combination was A1B1C3, which corresponds to an acrylic ester:epoxy resin ratio of 1:1, with TPGDA dosage accounting for 20% of the total system and photoinitiator dosage accounting for 5% of the total system, i.e., 1173:I-160 = 1:1.

Thus, a hybrid-UV cured photosensitive resin system consisting of 37.5% acrylic acid, 37.5% epoxy resin, 20% TPGDA, and 2.5% of both 1173 and I-160 was prepared based on the optimized formula obtained from the orthogonal analysis.

### 3.2. Structure Elucidation Analysis

**Figure 1:**
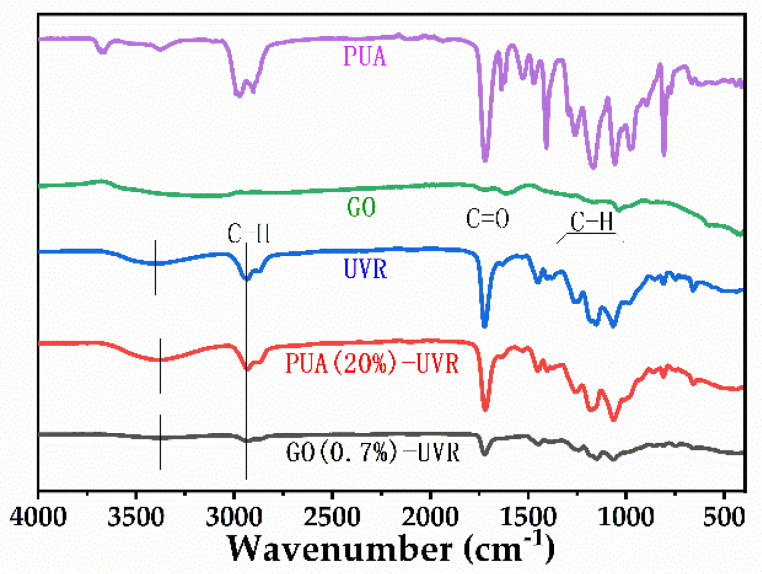
FTIR spectra of free−radical/cationic hybrid (UVR), polyurethane acrylates (PUA, 20%) and graphene oxide (GO, 0.7%) modified hybrid UV cured resins, PUA, and GO.

The FTIR spectra of the unmodified hybrid resin (UVR), 20% PUA- and 0.7% GO-containing hybrid UV cured resins, and PUA and GO from the literature are shown in Figure 1. The absorption peak at 3410 cm^−1^ is attributed to a hydroxyl group, which indicates the presence of the active diluent TPGDA or the occurrence of the ring−opening reaction of the epoxy resin. The shoulder peak at 2935 cm^−1^ is attributed to the saturated C−H bond, and the peak at 1720 cm^−1^ is attributed to the ester carbonyl group, which is part of the epoxy resin and exhibits a very strong absorption because it does not undergo any chemical reaction. The peaks at 1245 and 1150 cm^−1^ are attributed to the −C−O−C-stretching vibrations in the epoxy resin. Peaks corresponding to the stretching and bending modes of the unsaturated C-H appear at 1400 and 810 cm^−1^, respectively, but the characteristic peaks of the C=C double bond (1634 and 986 cm^−1^) were absent, as were those of the epoxy group (982, 910, and 772 cm^−1^). Therefore, we theorized that the epoxy resin in the hybrid system undergoes a complete ring-opening polymerization. After the photosensitive resin was completely cured, the FTIR spectra of PUA (20%)-UVR exhibits strong peaks at 3400 and 1720 cm^−1^ corresponding to the amino and carbonyl groups, respectively. However, the characteristic peaks of the terminal epoxy group at 750–900 cm^−1^ disappeared. These results imply that the ring-opening reaction of the epoxy resin was completed; however, a few unconverted double bonds remained in the system. Moreover, carbamate groups were present in the molecular structure. The FTIR spectra of GO (0.7%)-UVR shows a peak at 1720 cm^−1^ corresponding to the stretching vibration of the carbonyl group. The absence of any peaks characteristic of the C=C double bond or epoxy groups indicates that the curing reaction of the hybrid system was completed. The GO (0.7%)-UVR system exhibited a higher transmittance and lower absorption than the unmodified resin. Furthermore, the dispersion of GO into the colorless photosensitive liquid resin can block light absorption to some extent, owing to its yellowish brown color.

### 3.3. Hydrophobicity Analysis 

**Figure 2:**
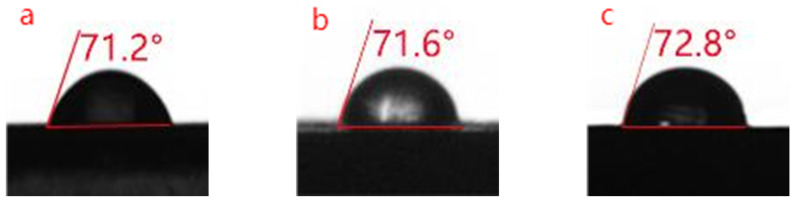
Water contact angles of (**a**) free−radical/cationic hybrid, (**b**) polyurethane acrylates (PUA, 20%) modified, and (**c**) graphene oxide (GO, 0.7%) modified photosensitive resins.

The water contact angle of the free−radical/cationic hybrid photosensitive resin at 25 °C measured in 1 s was 71.2° (Figure 2a), which is indicative of the wettability of the photosensitive resin and its tendency to absorb water. The water contact angle of the PUA-modified film was not significantly different (71.6°) from that of the unmodified film (Figure 2b), demonstrating the similarity in the wettability and water absorption properties of the two films. The GO (0.7%)-UVR curing film exhibited a water contact angle of 72.8° (Figure 2c), which is slightly larger than that of the unmodified resin. The contact angle of the materials with PUA and GO showed no significant difference from each other.

### 3.4. Morphological Analysis

**Figure 3:**
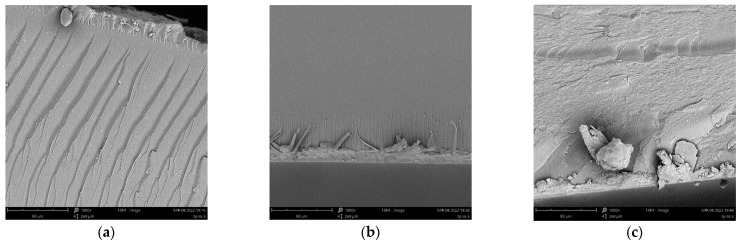
Scanning electron microscopy (SEM) images of (**a**) free radical −cationic/hybrid, (**b**) polyurethane acrylates (20%) modified, and (**c**) graphene oxide (0.7%) modified photosensitive resins in 1000× magnification.

Figure 3a shows that the cross section of the free−radical/cationic hybrid photosensitive resin is laminated, and the faults are uniform and orderly. This is because a small amount of uncured epoxy monomer in the resin acts as an impurity in the cured resin, resulting in the concentration of stress at the site of impurities during the fracture, thus forming stacking lines. The section of PUA-UVR in Figure 3b is smooth and flat without cracks or holes, indicating that it can form a relatively dense structure when it is lightly cured and stacked. The section of GO-UVR in Figure 3c is rough and uneven; however, the formation of agglomerates is not seen, indicating that GO is dispersed well in the resin. Furthermore, the section of GO-UVR that had high toughness, i.e., rough and uneven in the electron microscopy images, suggests that the addition of GO improves the toughness of the resin.

### 3.5. Thermogravimetric Analysis

**Figure 4:**
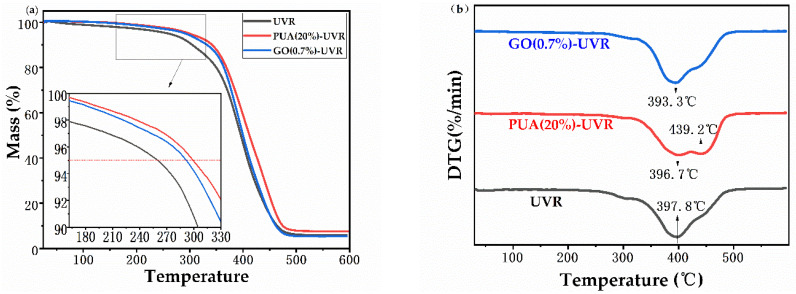
(**a**) Thermogravimetric (TG) and (**b**) Derivative Thermogravimetric (DTG) curves of free−radical/cationic hybrid photosensitive resin solidified product with polyurethane acrylates (20%) and graphene oxide (0.7%) modified photosensitive resins.

Owing to the difficulty in accurately determining the initial decomposition temperature of the material, the decomposition temperatures were taken as the temperatures at the corresponding weight losses of 5 wt% (Figure 4a). These temperatures and the residual weight at 500 °C were designated as T5 and W500, respectively. Upon introduction of PUA and GO, the initial decomposition temperature of the UV cured film gradually increased from 258.0 °C to 299.0 °C and 291.0 °C, respectively. Because PUA contains several C=C double bonds, it forms a denser crosslinking network upon curing, which renders the movement of chain segments more difficult, thus increasing the glass transition temperature. These results demonstrate that the addition of PUA and GO significantly improves the thermal stability of the polymer matrix. Additionally, the W500 of PUA (20%)-UVR was 7.85 wt% and that of the unmodified resin was 6.1 wt%. However, the W500 of GO (0.7%)-UVR was 6.21 wt%, owing to the existence of oxygen-containing functional groups on the surface of graphene oxide, which reduces the thermal stability of epoxy resin composites.

Derivative Thermogravimetric (DTG) curves show that UVR reaches the maximum thermal weight loss rate of 8.57% per min at 397.8 °C (Figure 4b). A total of 18.79% of the sample remained after thermal degradation. The DTG curve of PUA (20%) exhibits two weight loss peaks: the first weight loss occurs as a result of double bond breaking and the epoxy ring-opening, whereas the second may be attributed to the breaking of the C−N bond of the strongly polar carbamate group. Moreover, the temperature at which the maximum weight loss rate is observed increased in the PUA-UVR, demonstrating the improved heat resistance of the modified resin. The DTG curves of GO-UVR show similar maximum weight loss temperatures. The surface of GO contains a large number of oxygen-containing functional groups with relatively low thermal stability. A small amount of decomposition occurred at approximately 80 °C. Thus, the TG and DTG curves indicate that the thermal stabilities of both GO and PUA modified resins are similar. 

### 3.6. Crystallinity Analysis

**Figure 5:**
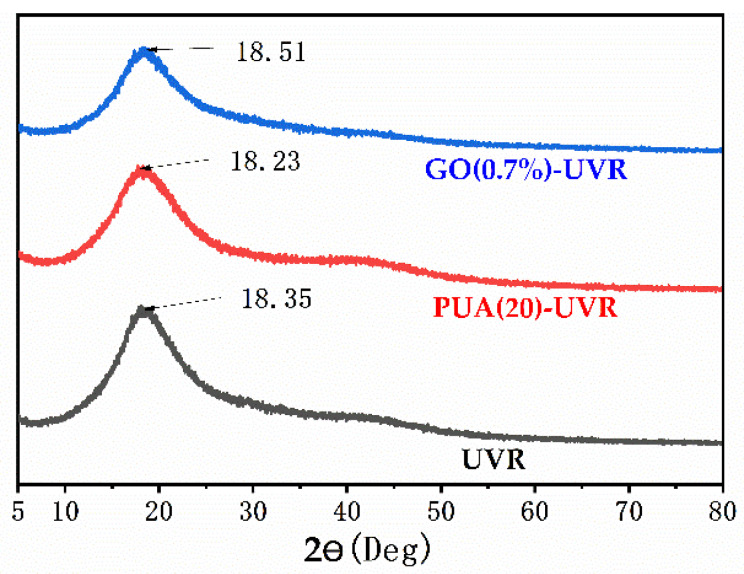
X-Ray Diffraction Spectra of free−radical/cationic hybrid (black), polyurethane acrylates (20%) modified (red), and graphene oxide (0.7%) modified (blue) photosensitive resins, respectively.

The crystallization behavior of cationic radical photosensitive resin and the effects of PUA and GO on the crystallinity of the resin were determined by X-ray diffraction (Figure 5). The spectra of all three resins show a wide dispersion peak at 2Ɵ = 18.4. The crystallization ability of PUA (20%)-UVR is weaker than that of UVR, and the crystallization ability of GO (0.7%)-UVR is weaker than that of PUA (20%)-UVR. This is due to the increase in the polymer cross−link generated by the modification of PUA and GO, which destroyed the symmetry and arrangement regularity of molecular chains.

### 3.7. Mechanical Properties Analysis

**Figure 6:**
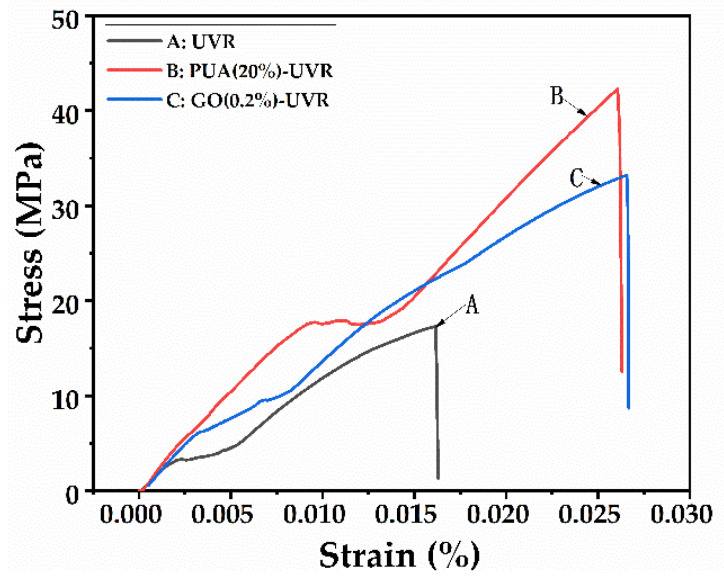
Tensile stress-strain of the free−radical/cationic hybrid, polyurethane acrylates (20%) modified, and graphene oxide (0.2%) modified photosensitive resins.

Figure 6 shows the tensile stress-strain plot of the resins. The mechanical properties of the resins were obtained by the tensile strength test, and the influence of the PUA and GO modifications on the mechanical properties of the free−radical/cationic hybrid photosensitive resin was explored via Young’s modulus (E) and tensile strength (σ). The tensile stress−strain plot suggests that all three resins possess brittle fracture, which is characteristic of a high crosslinking system. Additionally, the PUA and GO modifications result in a higher E and σ than those of the unmodified resin. The reason for the improvement of E and σ can be ascribed to the ultra-high molecular weight of PUA, which makes the molecular chain rigid, and the highly dispersed GO inorganic nanoparticles, which have strong interactions with a large number of polymer chains. The tensile strength of the resin with 10% PUA increased relative to the unmodified resin, reached a maximum (36.89 MPa) at 20% PUA, and then decreased to 28.31 MPa at 25% PUA (Table 6). When PUA, which forms free radicals, is added in excess to the resin, the free radical content of the system and the number of C=C double bonds rapidly increase. Therefore, the curing process is more susceptible to inhibition by oxygen, and, thus, the tensile strength of the resin is reduced. These results confirm that PUA enhances the mechanical properties of the photosensitive resin. At a GO:resin ratio of 0.2%, the tensile strength of the GO-UVR composite reached a maximum value of 29.27 MPa (Table 6). This value is 56% greater than that of the unmodified resin, which confirms the strengthening effect of GO. However, increasing the GO content has a significant detrimental effect on the tensile properties of the material owing to the agglomeration and poor dispersion of GO at higher mass fractions. The agglomerated graphene sheets act as defects in the composite material, which ultimately reduce the tensile strength of the material at GO mass fractions greater than 0.2%.

The Young’s modulus of the photosensitive resins decreased from 1820.12 MPa to 1726.12 MPa as the mass fraction of PUA increased from 0% to 15% PUA (Table 6), representing a reduction of 5.44% compared to that of the unmodified resin. The maximum Young’s modulus of 2295.14 MPa was observed at 20% PUA. These results indicate that 15% PUA has a significant toughening effect on the mechanical properties of the photosensitive resin. The GO-modified resin exhibited a similar trend. The Young’s modulus initially decreased as the GO mass fraction increased, reaching a minimum of 1549.99 at 0.2%. At a GO mass fraction of 0.5%, the Young’s modulus increased to 2642.22 MPa before reducing to 1712.90 MPa at 1% GO. Nevertheless, the toughening effect of 0.2% GO on the photosensitive resin is evident (Table 6).

### 3.8. Volume Shrinkage Rate Analysis

**Figure 7:**
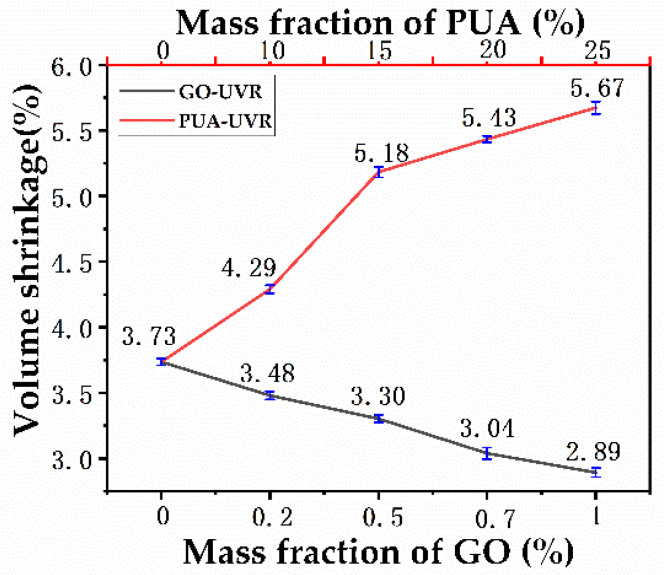
Volume shrinkage of free−radical/cationic hybrid, polyurethane acrylates modified, and graphene oxide modified photosensitive resins.

Polymerization of the resin involves a transformation from short−chain small molecules to long−chain macromolecular polymers, which greatly alters the molecular structure of the material. Figure 7 shows a plot of mass fraction vs. volume shrinkage. We observed that volume shrinkage gradually increased with an increase in the PUA content in the resin. The maximum volume shrinkage of 5.67% was observed in PUA (20%)-UVR, which is primarily attributed to the change in bonding from the longer length intermolecular van der Waals attractions to the shorter length covalent bonds. Higher mass fractions of PUA in the resin result in higher free radical content in the system and thus in greater volume shrinkage, owing to the high molecular weight and large number of functional groups in PUA. Volume shrinkage of the GO-modified resin system decreased with increasing GO content. GO (0.2%)-UVR underwent a volume shrinkage of only 3.48, whereas GO (1%)-UVR exhibited an even lower volume shrinkage of 2.89. This may be because GO does not participate in chemical reactions but rather acts as an inorganic filler owing to its wide and even dispersion. Therefore, a moderate GO content is required, because an excessive amount will affect the gelation rate and mechanical properties of the resin.

### 3.9. Gelation Rate Analysis

**Figure 8:**
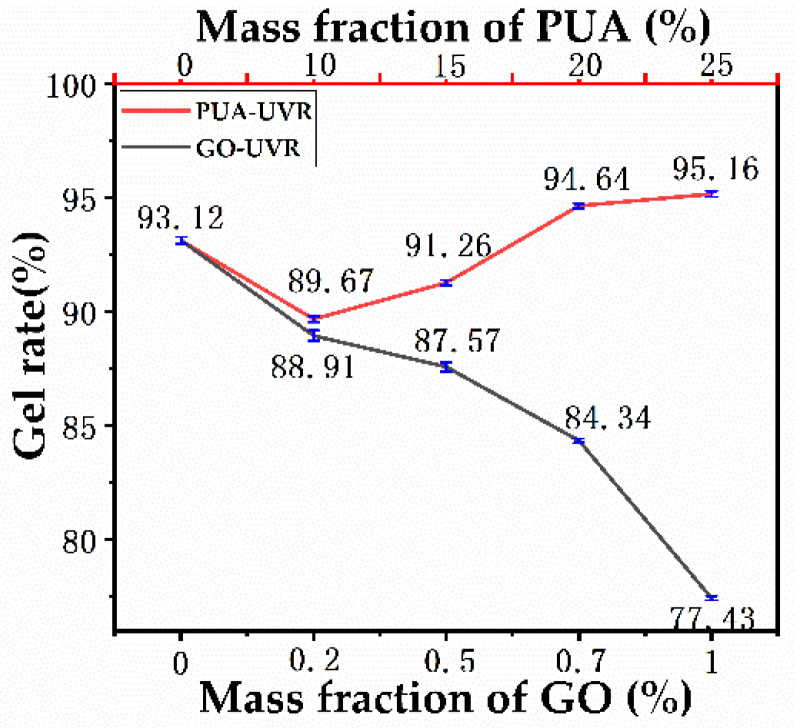
Gelation rate of free−radical/cationic hybrid, polyurethane acrylates modified, and graphene oxide modified photosensitive resins.

Figure 8 indicates that the gelation rates of the unmodified hybrid photosensitive resin (93.12%) and 10% PUA (89.67%) modified resin were the highest and lowest, respectively. The ultra-high molecular weight of PUA limits the movement of the polymer chain, thereby weakening or even offsetting the crosslinking density and thus affecting the gelation rate. In addition, the number of active double bonds in the system increased with the mass fraction of PUA. Therefore, increasing the mass fraction of PUA can increase the gelation rate, which reached a maximum of 95.16% at 25% PUA. Conversely, increasing the mass fraction of GO resulted in a reduction of the gelation rate of the modified resin. At 0.2% GO content, the gelation rate decreased from 93.12% to 88.91% and further decreased with increasing GO content (0.5% and 0.7%). At 1% GO content, the gelation rate reached the minimum of 77.43%. GO is an opaque carbon filler, which does not react with the photosensitive resin but exists as a neutral independent phase. Upon polymerization and curing of the photosensitive resin, GO hinders the movement of the polymer chain, thereby hindering further polymerization; thus, it inhibits the gelation rate, resulting in slow curing inside the photosensitive resin.

### 3.10. Rheological Properties Analysis

**Figure 9:**
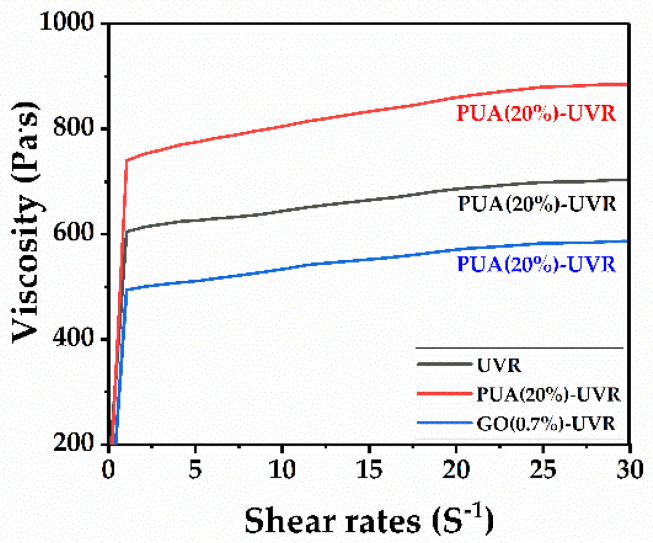
Viscosity relation of free−radical/cationic hybrid, polyurethane acrylates (20%) modified, and graphene oxide (0.7%) modified photosensitive resins.

Viscosity is an extremely important property of photosensitive resins and directly reflects their fluidity. The rheological properties of the photosensitive resin sample before light curing are shown in Figure 9. The viscosity of the PUA−modified resin is high owing to its functionality and relative molecular weight being 6 and 1160, respectively. The addition of PUA increases the relative molecular weight and viscosity of the resin. GO with a lamellar structure is dispersed in the photosensitive resin system, which reduces the intermolecular force and increases the intermolecular distance. Therefore, the viscosity of the resin is reduced after GO modification. Additionally, GO modification provides the molding strength for light curing printing.

## 4. Conclusions

A UV curable free−radical/cationic hybrid photosensitive resin was prepared by the polymerization of free radical and cationic oligomers in the presence of active diluents and photoinitiators. An optimal acrylate to epoxy resin ratio of 1:1 was determined. The tensile strength of the resin was significantly improved by the addition of PUA, reaching the maximum of 36.89 MPa at a PUA content of 20%. Moreover, increasing the mass fraction of GO in the system reduced the volume shrinkage, which reached the minimum of 2.89% at a GO content of 1%. Our future work in this area aims to develop photosensitive resins with improved tensile strength and reduced volume shrinkage for applications in high-performance functional materials.

## Figures and Tables

**Table 1:** The orthogonal experimental factors and levels.

	Level	A	B/%	C/%
Factors				
1		1:1	20	3
2		2:1	30	4
3		3:1	40	5

**Table 2:** The orthogonal experimental scheme.

	A	B/%	C/%	Null Columns
①	1:1	20	3	1
②	1:1	30	4	2
③	1:1	40	5	3
④	2:1	20	4	3
⑤	2:1	30	5	1
⑥	2:1	40	3	2
⑦	3:1	20	5	2
⑧	3:1	30	3	3
⑨	3:1	40	4	1

**Table 3:** Results of the orthogonal experiments.

A	B/%	C/%	Null Columns	Tensile Strength/MPa	Volume Shrinkage/%	Gel Rate/%
1:1	20	3	1	17.35	3.81	87.39
1:1	30	4	2	19.44	4.87	90.11
1:1	40	5	3	15.58	6.38	88.65
2:1	20	4	3	16.23	4.52	89.26
2:1	30	5	1	18.50	5.30	95.28
2:1	40	3	2	14.90	6.96	84.53
3:1	20	5	2	15.05	5.44	90.72
3:1	30	3	3	11.80	6.53	83.70
3:1	40	4	1	10.34	7.3	85.14

**Table 4:** Range analysis results.

		A	B	C	Null Columns	Primary and Secondary Order	Optimum Combination
Tensile strength	k1	17.46	16.21	14.68	15.4	A > B > C	A1B2C3
	k2	16.54	16.58	15.34	16.46		
	k3	12.4	13.61	16.38	14.54		
	R	5.06	2.97	1.69	1.93		
Volume shrinkage	k1	5.02	4.59	5.77	5.49	B > A > C	A1B1C2
	k2	5.59	5.57	5.58	5.76		
	k3	6.44	6.9	5.71	5.81		
	R	1.42	2.31	0.18	0.32		
Gel rate	k1	88.72	89.12	85.21	89.27	C > B > A	A2B2C3
	k2	89.69	89.7	88.17	88.45		
	k3	86.52	86.11	91.55	87.2		
	R	3.17	3.59	6.34	2.07		

**Table 5:** Statistical analysis of the orthogonal experiments.

		Sum of Squares of Deviation	Degrees of Freedom	The Variance	F	Fa	Significant Level
Tensile strength	A	43.633	2	21.8165	7.806	19	F < Fa
	B	15.755	2	7.8775	2.818	19	F < Fa
	C	4.376	2	2.188	0.783	19	F < Fa
	error	5.59	2	2.795			
	sum	69.354					
Volume shrinkage	A	3.077	2	1.5385	17.094	19	F < Fa
	B	8.068	2	4.034	44.822	19	F > Fa, remarkable
	C	0.052	2	0.026	0.289	19	F < Fa
	error	0.18	2	0.09			
	sum	11.377					
Gel rate	A	15.822	2	7.911	2.434	19	F < Fa
	B	22.317	2	11.1585	3.433	19	F < Fa
	C	60.444	2	30.222	9.299	19	F < Fa
	error	6.5	2	3.25			
	sum	105.083					

**Table 6:** Tensile strength and Young’s modulus of the unmodified and modified hybrid resins.

	Tensile Strength (MPa)	Young’s Modulus (MPa)
UVR	16.42	1820.62
PUA (10%)-UVR	28.76	1893.24
PUA (15%)-UVR	32.11	1726.12
PUA (20%)-UVR	36.89	2296.14
PUA (25%)-UVR	28.31	2128.34
GO (0.2%)-UVR	29.27	1549.99
GO (0.5%)-UVR	13.16	2642.22
GO (0.7%)-UVR	11.84	2597.72
GO (1%)-UVR	12.07	1712.90

## Data Availability

The data presented in this study are available on request from the corresponding author.

## Supplementary Materials

The following supporting information can be downloaded at: https://www.mdpi.com/article/10.3390/polym14101959/s1. Figure S1: SEM of Powdered GO.
