# Supplementary figures and images for: Synthesis and Optimization of a Free-Radical/Cationic Hybrid Photosensitive UV Curable Resin Using Polyurethane Acrylate and Graphene Oxide
Source: Polymers (Basel). 2022 May 12;14(10):1959. doi: 10.3390/polym14101959 (PMC9145890; doi:10.3390/polym14101959)

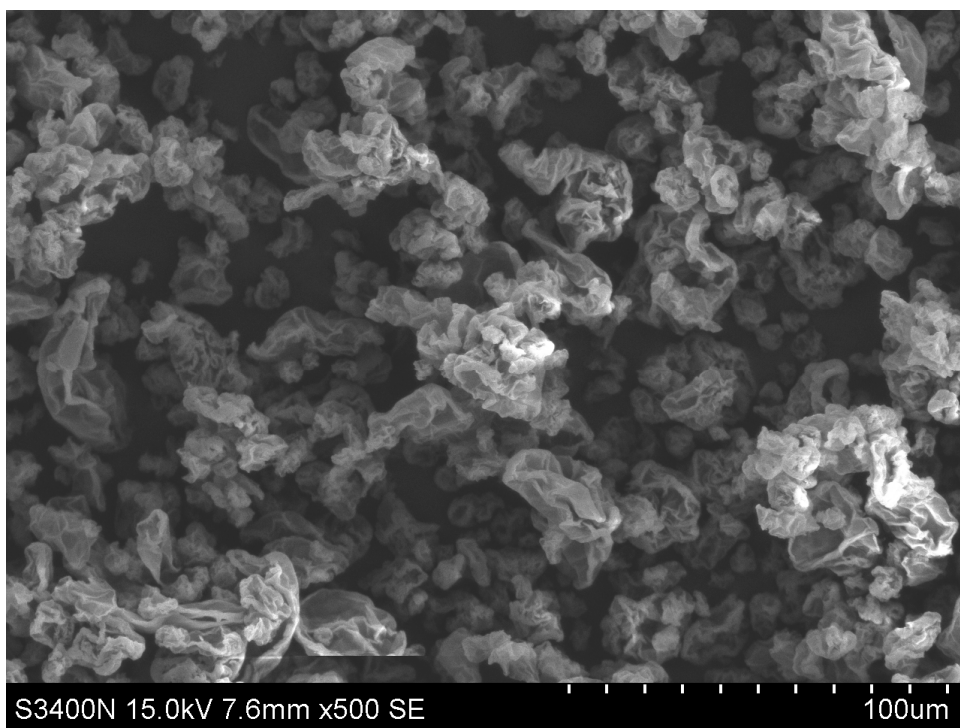

**Figure S1.** SEM of Powdered GO.

Supplement: Supplementary file 1 [file polymers-14-01959-s001.zip › polymers-1353533-supplementary.pdf]
